# McArdle Disease vs. Stiff-Person Syndrome: A Case Report Highlighting the Similarities Between Two Rare and Distinct Disorders

**DOI:** 10.3389/fneur.2020.529985

**Published:** 2020-11-10

**Authors:** Kerilyn Godbe, Giovanni Malaty, Alyssa Wenzel, Sahana Nazeer, Douglas J. Grider, Adrienne Kinsey

**Affiliations:** ^1^Virginia Tech Carilion School of Medicine, Roanoke, VA, United States; ^2^Department of Basic Science, Virginia Tech Carilion School of Medicine, Roanoke, VA, United States; ^3^Department of Family Medicine, Carilion Clinic, Roanoke, VA, United States

**Keywords:** McArdle disease, Stiff Person Syndrome, case report, autoimmune disease, diagnostic workup

## Abstract

McArdle disease is a rare autosomal recessive disorder of muscle glycogen metabolism that presents with pain and fatigue during exercise. Stiff-Person Syndrome is an autoimmune-related neurologic process characterized by fluctuating muscle rigidity and spasm. Reported is a 41-year-old male who presented to the emergency department due to sudden-onset weakness and chest pain while moving his refrigerator at home. Cardiac workup was non-contributory, but a creatine kinase level > 6,000 warranted a muscle biopsy. The biopsy pathology report was misinterpreted to be diagnostic for McArdle disease given the clinical presentation. After 4 years of treatment without symptomatic improvement, a gradual transition of symptoms from pain alone to pain with stiffness was noted. A positive glutamic acid decarboxylase antibody test resulted in a change of diagnosis to Stiff-Person Syndrome. This is the first known case that highlights the similarities between these two rare and distinct disease processes, highlighting the necessity for thorough history taking, maintenance of a broad differential diagnosis, and knowledge of how best to interpret complex pathology reports.

## Introduction

Inherited disorders of glycogen storage and metabolism are rare, with an annual incidence of one case per 20,000–25,000 births in the United States (1). Myophosphorylase deficiency, also called glycogen storage disease type V, or McArdle disease, is an autosomal recessive disorder on chromosome 11q13 that causes mutations in muscle glycogen phosphorylase, an allosteric enzyme that contributes to the metabolism of glycogen to glucose-1-phosphate. Dysfunction of myophosphorylase causes downstream ATPase failure resulting in exercise intolerance, myalgias, myoglobinuria, and elevated resting creatine kinase. Diagnosis is made via a history that includes recurrent episodes of exercise-induced symptoms, PYGM next-generation DNA sequencing (NGS) panel for myophosphorylase, cycle-ergometer testing, or muscle-section biopsy and histochemical staining for myophosphorylase. Diagnosis of McArdle disease is regularly delayed by years following initial presentation, and can be misdiagnosed in up to 90% of adolescents and young adults. Mistreatment is common, with alternate diagnoses regularly including lack of physical fitness, rheumatic disorders, psychological disturbance, and neuromuscular disease (2).

Stiff-Person Syndrome (SPS) is a gradually-progressing neuromuscular disorder in which the autoimmune-mediated blockade of the central nervous system (CNS) inhibitory enzyme glutamic acid decarboxylase (GAD) causes progressive axial muscle stiffness and rigidity (3). Annual incidence is approximately one case per million in the United States, with 20–50 year old men being the most commonly affected population (4, 5). The autoimmune component of SPS is derived from the disease's frequent association with other autoimmune disorders such as type 1 diabetes mellitus, thyroiditis, and vitiligo (6). Unlike McArdle disease, there is no proposed hereditary association, therefore no genetic testing is available. Diagnosis of SPS involves a primary index of suspicion based on recurrent symptomatic presentation involving axial muscle stiffness and superimposed episodic spasms. Approximately 60–80% of individuals with SPS will have CSF anti-GAD65 antibody (a 65 kDa isoform of GAD) titers that exceed 1,000 units/mL, though it is unknown whether these antibodies are pathogenic. Even with diagnosis and treatment, the prognosis remains variable and unpredictable.

## Case Description

A 41-year-old man with a history of hypertension and type 2 diabetes mellitus presented to the emergency department with cramping chest pain, sudden-onset weakness, and loss of consciousness while moving his refrigerator at home. At this time, the patient had over fifteen prior office visits in which he complained of muscle weakness and stiffness of his legs, back, and neck following any physical exertion. This included workout warm up, and even upon waking up in the morning with no identifiable cause. Patient was treated for these episodes with antispasmodics and physical therapy, and his differential diagnosis included muscle strain and muscle spasm. During these episodes, questions about the “second wind” phenomenon were not recorded. Cardiac workup was non-contributory, but a creatine kinase and aldolase levels were noted to be > 6,000 U/L (nl 22–198 U/L) and 40.0 U/L (nl 1.0–7.5 U/L), respectively. He was treated for acute rhabdomyolysis and his creatine kinase normalized. He subsequently underwent further diagnostic laboratory testing including muscle biopsy of the left quadriceps. Erythrocyte sedimentation rate (ESR) and C-reactive protein (CRP) levels were normal, and an anti-Jo antibody panel for polymyositis, a proposed diagnosis at that time, was negative. Hematoxylin and eosin (H&E) stain of the left quadriceps muscle biopsy showed the presence of normal muscle fibers with no evidence of perifascicular atrophy or endomysial inflammation. A panel of special histochemical stains showed no evidence of fibrosis, red-ragged fibers or abnormal lipids. However, a Periodic acid-Schiff (PAS) stain revealed slight staining uptake around the periphery of some of the fibers, suggesting the possibility of glycogen storage disease. However, no massive accumulation of glycogen was seen, as is usually the case in McArdle disease. Electron microscopy showed prominent excessive glycogen accumulation within both the subsarcolemmal areas and free within the cytoplasm. Electromyographic testing was suggestive of a chronic myopathic process without evidence of myonecrosis or vasculitis. These results, in addition to history of elevated creatine kinase and muscle aches following exercise, were interpreted to support McArdle disease. At this time, the patient elected to initiate care at a larger, more specialized institution.

For the next 2 years, the patient suffered recurrent falls with increasing frequency of visits to the emergency department for muscle cramps and spasms, insidiously causing posturing of his trunk and limbs. He had elevated aldolase and creatine kinase levels on a semi-continuous basis. He was hospitalized several times given the severity of his symptoms, and often received high doses of benzodiazepines in addition to intubation due to fear of spasms causing acute respiratory crisis. His pain was treated with a baclofen pump, hydromorphone, and various other intravenous narcotics—none of which were reported to alleviate his pain. Progressive visits gradually transitioned from complaints of muscle pain and fatigue to cramps with spasms and stiffness, prompting alternative diagnostic testing, including a GAD65 antibody level, which was undetectable. Shortly thereafter, the patient agreed to genetic testing, not previously performed, as management would have been unchanged with a positive result. Testing yielded a negative myopathy/rhabdomyolysis NGS panel in which only one variant was found in the ACADVL gene out of all 27 total genes tested; the clinical significance of which is currently unclear. The patient was not found to have a PYGM mutation. At this point, the patient elected once again to transition his care to a more specialized institution for an alternate diagnostic opinion. On specific questioning at that institution, it was discovered that the patient was developing urinary hesitancy, cognitive slowing, insomnia, irritability, frequent headaches, and difficulty swallowing solids. This institution questioned his diagnosis of McArdle disease, and repeat muscle biopsy was recommended, which showed type-2 muscle fiber predominance, fiber regeneration, mild increases in lipid and glycogen, and non-specific mitochondrial changes, not dissimilar from his initial muscle biopsy findings. At this time etiologic considerations included glycogen storage disorder, mitochondrial disorder, or adult onset muscular dystrophy. There was no immediate suggestion of an immune-mediated inflammatory myopathy, and his treatment continued unchanged.

**Figure 1:**
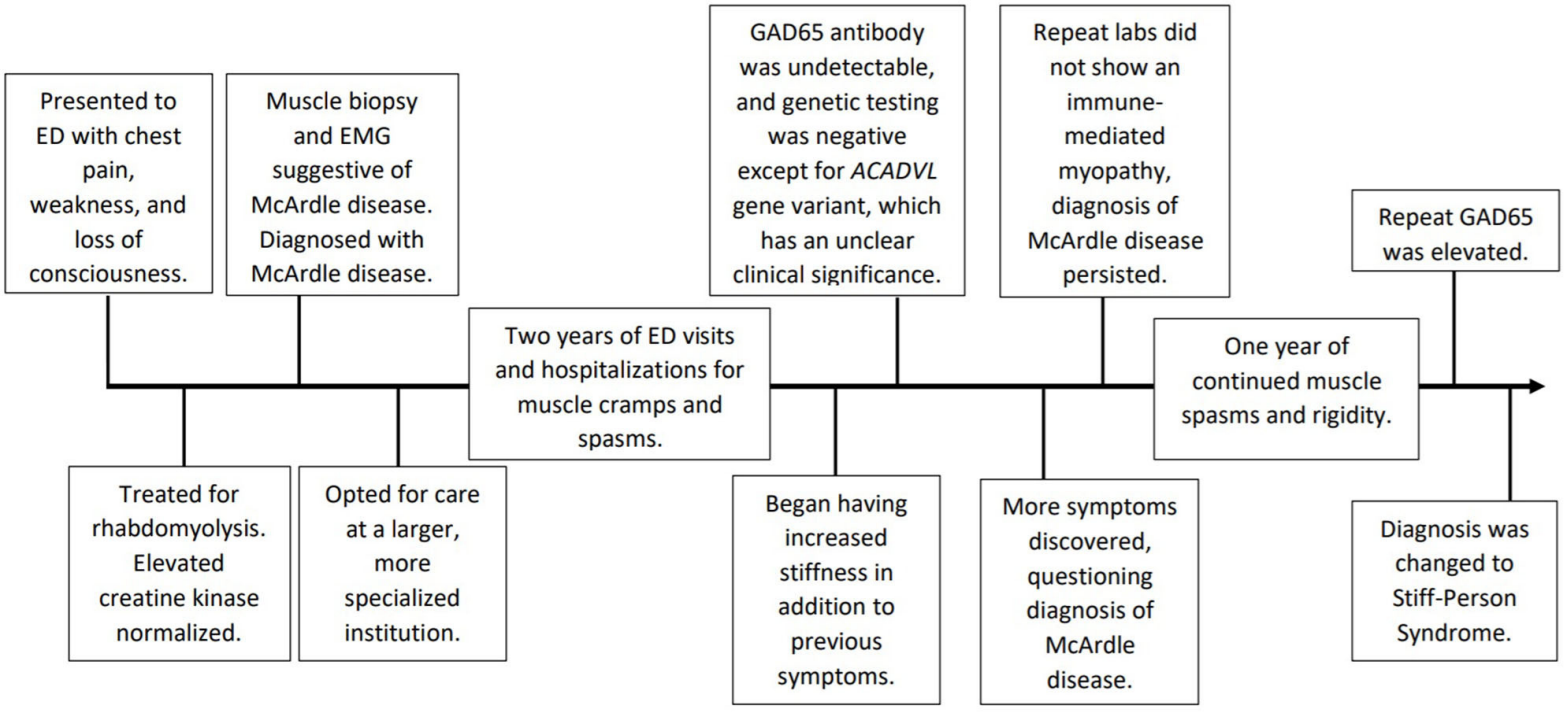
Chronological depiction from patient's initial presentation to final diagnosis.

Approximately 1 year later at a follow-up appointment with continued symptoms, the patient was tested again for GAD65 antibodies which yielded an elevated level at 76.0 nmol/L (nl≤0.02 nmol/L). This level resulted in a change in his initial diagnosis from McArdle disease to Stiff-Person Syndrome. See Figure 1 for a chronological depiction of his complex diagnostic course. Therapy was started accordingly. Today, the patient is 48 years old and has tried various treatment regimens due to persistent symptoms. The patient has been hospitalized twice every month for the past 4 months. The patient is currently using an implanted baclofen pump and diazepam to help control his spasms. He has a hydromorphone implanted pump for pain. For treatment of his disease, the patient is currently taking mycophenolate 500 mg twice a day and prednisone 10 mg every other day. Prior regimens included two sessions of port-administered plasmapheresis every 2–3 weeks, which was not successful and discontinued. A trial of intravenous immunoglobulin daily for 2 days every 3 weeks did not provide symptom relief. Rituximab was tried several years ago on a 4–6 months interval and was also ineffective.

While the patient persistently undergoes routine hospitalization for similar symptoms, his anti-GAD65 antibody level has returned to normal. Recently, a serum paraneoplastic antibody evaluation (ANNA-1, ANNA-2, ANNA-3, AGNA-1, ACh, AChR, VGKC, PCA-1, PCA-2, PCA-Tr, CRMP-5, and Amphiphysin) was performed, and no abnormalities were detected. Understandably as time passed, the patient became more frustrated with the lack of answers provided by the multitude of physicians. His symptoms dated back to his adolescence and as the process started, he began bouncing between multiple medical centers without any progress toward a definitive diagnosis. Despite the extensive testing, the patient was continuing to have symptoms with no relief from the treatments, leading to his growing dissatisfaction with the process.

The patient's persistent symptoms prompted an extensive chart review, which revealed a personal history of various non-descript musculoskeletal, neurological, and respiratory complaints. Since age 16, the patient was seen for possible Cystic Fibrosis (CF) workup, intermittent headaches, muscle strains and spasms following light exercise, acute episodes of respiratory failure, and depression. A history of childhood trauma with subsequent psychiatric difficulty was at one point considered to be contributory to his complex array of symptoms. One prevailing notion held by a select few of his previous physicians is that his symptoms represented a functional neurological/psychiatric disorder, not related to a defect in glycogen-storage. A review of his family history was negative for any members with similar symptoms at any age. Given that this patient was diagnosed with two rare diseases that have variable presentation and progression that are not entirely clear, no adverse or unanticipated events have been recorded other than repeat hospitalizations despite treatment.

## Discussion

This patient's diagnostic history features a progression from one rare and unique pathology to another, each with its own pathological basis, diagnostic criteria, and management strategy. This progression highlights the importance of thorough history-taking, distinguishing similar presentations such as pain, spasm, and rigidity, and knowledge of what diagnostic tests exist for rare metabolic and autoimmune disorders of the musculature. This case presents unique diagnostic challenges for healthcare providers at facilities that may not routinely manage complex pathologies that involve genetic or autoimmune diagnostic testing. The combination of rarity and limited management options for these disorders, if a diagnosis is reached, makes each of them difficult to manage. Pursuing nuanced diagnostic information such as obtaining genetic testing or performing a cycle-ergometer test may be out of the scope or resources of a care facility seeing a patient with this presentation. Similarly, questioning about the second wind phenomenon may not be apparent to providers who are unfamiliar with working up these diseases. We urge providers who are unfamiliar with the workup of rare genetic diseases, or those at facilities with limited resources, to be wary or presentations that include vague symptoms such as pain and fatigue.

A thorough review of the current literature yielded no recorded cases of an individual having been worked up for both diseases in their lifetime. With this lack of background information in addition to the fact that both McArdle disease and Stiff-Person Syndrome are exceedingly rare on their own, this case report is limited in its ability to determine what is the best diagnostic work-up, the most efficacious treatment, nor can it be determined the etiology of the progression from McArdle to Stiff-Person Syndrome. However, with the patient's nearly complete medical record at our disposal, this case report was able to take an in-depth look at how potential patients can present and what medical providers are doing to work up vague complaints like fatigue, muscle pain, and cramping.

Differentials for his extensive history of musculoskeletal complaints included muscle spasms, ordinary muscle strain, and a negative workup for rheumatoid arthritis, featuring no arthritic changes on x-ray. After his first hospitalization, the main differential diagnoses included overexertion and possible polymyositis. Both diagnoses were eliminated due to persistence of these complaints and negative polymyositis workup. Only after a muscle biopsy was the possibility of McArdle disease introduced. As this patient did, individuals with McArdle disease typically present as adolescents or young adults with poor endurance, fatigue, muscle cramping, and weakness following periods of isometric or prolonged dynamic exercise. The immediate cause may not be apparent at presentation. The “second wind” phenomenon is also a frequently reported symptom of McArdle disease, characterized by the ability to resume exercise with less difficulty after a short period of rest (7). In 2017, Santalla et al. (7) reported that although most patients show the second wind phenomenon, 21% of patients the 239 patients studied showed limitations during daily activities and fixed muscle weakness, as in this patient. It was not recorded if this patient was asked about the “second wind” phenomenon during his initial workup, highlighting the difficulty of working up a rare disease by physicians who are not familiar with the steps and management. Patients may also present as older adults, however, exhibiting similar symptoms in addition to more regularly-reported episodes of myoglobinuria, which has the potential to result in acute kidney injury in 4–11% of patients (8, 9). Although this patient had persistently elevated CK, myoglobinuria was not frequently reported. The two genetic forms of myophosphorylase deficiency that have been identified, childhood-onset and adult-onset, are both autosomal recessive mutations of PYGM gene on chromosome 11q13. Within these forms, ~95 different genetic mutations have been observed, though there have been no identifiable genotypic-phenotypic correlates; and carriers of the mutation are asymptomatic (10–12). In histochemical analysis of frozen muscle biopsies of individuals with McArdle disease, little to no myophosphorylase activity, and increased perifibrous glycogen accumulation will be observed, though post-rhabdomyolysis regenerating muscle specimens may exhibit normal enzymatic activity, leading to false positives (13). The patient's initial diagnosis was made on symptomatic history and misinterpretation of his muscle biopsy findings, with genetic testing deferred for 3 years due to cost and lack of influence on subsequent management. Eventual genetic testing showed no mutation of the PYGM gene. Management of McArdle disease includes diet modification with increased-carbohydrates, as well as increasing exercise-tolerance with regular, moderate aerobic exercise. It was not apparent from the patient's chart or history whether these recommendations were made during his initial and subsequent disease management.

Following the transition of the patient's symptoms from muscle-aches to increased stiffness and spasms, and in the light of a positive repeat GAD65-Ab test, the diagnosis was changed to Stiff Person Syndrome. Interestingly, the positive result came nearly 2 years after an initial GAD65 test was negative. Subsequent repeated tests have also been negative, presumably due to initiated treatment. Repeat muscle biopsy showed no evidence of any autoimmune process, which is the proposed pathogenic basis of Stiff-Person Syndrome. While there exists some demonstrated association between anti-GAD65 antibodies and Stiff-Person Syndrome, up to 30% of patients may be antibody-negative. Further, the specific role of the GAD antibody in the pathogenesis of the disease remains unclear (14). Several subtypes of Stiff-Person Syndrome exist which are differentiated based on their association with specific antibody isoforms and other autoimmune processes. All are GAD-positive, and include Classic (generalized stiffness and spasms), Partial (unilateral symptoms), and Paraneoplastic (clinically similar to classic but involves various other antibodies) Stiff-Person Syndrome. The patient's non-contributory paraneoplastic panel was administered to rule out this subtype. In addition to symptomatic history and a positive GAD-antibody test, diagnosis may involve electromyographic (EMG) testing and therapeutic diazepam trial (15). EMG testing was suggestive of a chronic myopathic process without evidence of myonecrosis or vasculitis. Benzodiazepines were frequently used for partial symptomatic relief during hospitalizations when the diagnosis was believed to be McArdle disease. The patient is currently taking therapeutic diazepam. Differential diagnosis for Stiff-Person Syndrome is diverse including neurological, autoimmune, and infectious disorders like Parkinsonism, axial dystonia, Ankylosing Spondylitis (AS), and tetanus (16). Luckily, and presumably due to his already-extensive history, this patient was not subjected to a long, unnecessary workup for any of these listed differential diagnoses. Standard treatment for Stiff-Person Syndrome mirrors this patient's treatment regimen closely, and consists of regular plasma exchange, clonazepam, rituximab, and as-needed baclofen. Prognosis of Stiff-Person Syndrome remains variable, and while strong pharmacologic treatment may prolong ambulation, a majority of patients will develop physical ambulatory disability (6). This likely-inevitable physical decline will be monitored in this patient.

While these two distinct and rare disease processes have been the main diagnoses in this patient's case, neither has been an ideal diagnostic fit, and it is possible that the correct diagnosis has yet to be identified. Further, it is unknown whether this patient's psychiatric history plays a role in his current or past states of health. This is yet one additional obfuscating factor in identifying a definitive diagnosis. While treatment for McArdle disease seemed ineffective, his current treatment for the diagnosis of Stiff-Person Syndrome has demonstrated episodic improvement in his stiffness and laboratory improvement of his antibody levels. In a case series consisting of 57 patients, Rokocevic et al. (17) reported that levels of GAD antibody varied over time, with no correlation to disease severity. Although the anti-GAD levels of these 57 patients varied, all remained persistently elevated, unlike the patient in this case, adding another level of complexity (17). Regardless of whether the correct diagnosis has already been—or will 1 day be made, this patient's case presents many nuanced diagnostic challenges, reminding us of the importance of thorough history-taking and broad laboratory acumen, including the importance of knowing how to interpret complex pathology reports.

## Ethics Statement

Written informed consent was obtained from the individual for the publication of any potentially identifiable images or data included in this article.

## Author Contributions

KG performed the patient's chart review, transcribed the case, and edited the manuscript. GM authored the discussion and edited the manuscript. AW edited the manuscript, prepared it for submission, and presented the case. SN edited the manuscript and prepared it for submission. DG reviewed the pathology results and interpretation and edited the manuscript. AK approved of the case and provided oversight of the project, as the patient has been under AK's care for over five years. All authors contributed to the article and approved the submitted version.

## Conflict of Interest

The authors declare that the research was conducted in the absence of any commercial or financial relationships that could be construed as a potential conflict of interest.
